# A New Cryptic Species of South American Freshwater Pufferfish of the Genus *Colomesus* (Tetraodontidae), Based on Both Morphology and DNA Data

**DOI:** 10.1371/journal.pone.0074397

**Published:** 2013-09-11

**Authors:** Cesar R. L. Amaral, Paulo M. Brito, Dayse A. Silva, Elizeu F. Carvalho

**Affiliations:** 1 Department of Zoology, Universidade Estadual do Rio de Janeiro, Rio de Janeiro, Brazil; 2 Department of Ecology, Universidade Estadual do Rio de Janeiro, Rio de Janeiro, Brazil; Tel-Aviv University, Israel

## Abstract

The Tetraodontidae are an Acantomorpha fish family with circumglobal distribution composed of 189 species grouped in 19 genera, occurring in seas, estuaries, and rivers between the tropical and temperate regions. Of these, the genus *Colomesus* is confined to South America, with what have been up to now considered only two species. *C. asellus* is spread over the entire Amazon, Tocantins-Araguaia drainages, and coastal environments from the Amazon mouth to Venezuela, and is the only freshwater puffers on that continent. *C. psittacus* is found in coastal marine and brackish water environments from Cuba to the northern coast of South America as far south as to Sergipe in Brazil. In the present contribution we used morphological data along with molecular systematics techniques to investigate the phylogeny and phylogeography of the freshwater pufferfishes of the genus *Colomesus*. The molecular part is based on a cytochrome C oxidase subunit I dataset constructed from both previously published and newly determined sequences, obtained from specimens collected from three distinct localities in South America. Our results from both molecular and morphological approaches enable us to identify and describe a new *Colomesus* species from the Tocantins River. We also discuss aspects of the historical biogeography and phylogeography of the South American freshwater pufferfishes, suggesting that it could be more recent than previously expected.

## Introduction

The Tetraodontidae is an Acantomorpha fish family with circumglobal distribution composed of 189 species in 19 genera, occurring in seas, estuaries, and rivers between the tropical and temperate regions [1]. They are mainly characterized by their typical four large dental plates; the ability to inflate their body in stressful situations; the presence of the neurotoxin Tetrodotoxin/Saxitoxin in its tissues, being responsible for numerous cases of fatal poisoning in many countries, including Brazil; and by having the smallest genome among vertebrates, therefore being considered as a model for the genome evolution of the group.

Among the Amazonian taxa exploited by the ornamental fish industry in South America are those of *Colomesus* [2], a genus confined to South America, with what is presently considered two species, *C. asellus* and *C. psittacus*. *C. asellus* [3] is spread in the entire Amazon, Tocantins-Araguaia drainages, and coastal environments from the Amazon mouth to Venezuela, being the only freshwater puffers on that continent. *C. psittacus* [4] is found in coastal marine and brackish water environments from Cuba and the northern coast of South America to Sergipe in Brazil.

Mainly located in tropical and subtropical regions all around the world, including the Amazon region, the ornamental fish industry is one of the largest transporters of live animals and plants with an annual trade volume estimated at U$15–25 billion [5]–[7], in a scenario where species identification problems, mainly related to border biosecurity are not rare.

The DNA barcode is a widely accepted tool for species determination mainly due to its enhanced attention on standardization and data validation [8], being a rapid and low cost method of identification [9]. The use of DNA barcoding techniques has been utilized in many taxa, including bacteria, birds, bivalves, butterflies, fishes, flies, macroalgae, mammals, spiders, sprigtails, and also for plants [10]–[27].

The DNA barcode technique for Metazoans uses a short (∼650 bp) and standardized gene region from the mitochondrial 5′ region of the cytochrome C oxidase subunit I (COI) for a rapid and cost-effective animal identification. This has been demonstrated to be an effective fish identification tool in numerous situations, including consumer protection [28]–[30], fisheries management/conservation [31], border biosecurity in the ornamental fish trade [5], and in the identification of overlooked or cryptic species [32].

Here we used both morphological and molecular methodologies in an integrative taxonomical approach to investigate the diversity of the Amazonian freshwater pufferfishes of the genus *Colomesus* based on specimens collected from three distinct populations from both Brazil and Peru. Additionally, we describe a new *Colomesus* species from the Upper Tocantins drainage based on both morphological and molecular data.

## Methods

**Figure 1:**
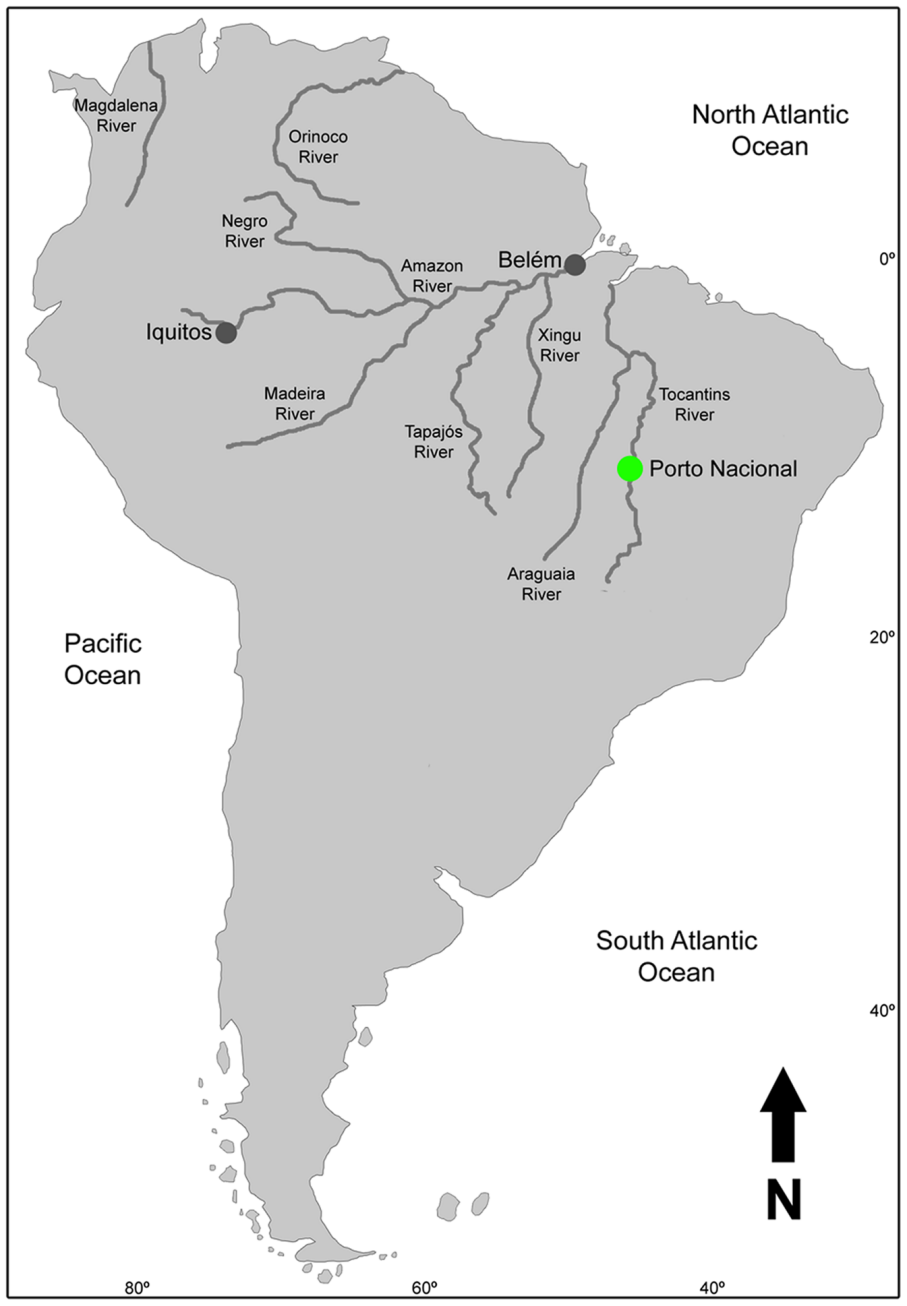
Map of South America showing the northern hydrology and the localities where the specimens were collected (grey and green marks).

Specimens of *Colomesus asellus* were collected from three distinct populations with about 2200 km of mean distance separating them. The collection localities were Ilha do Mosqueiro, Belém, Brazil; Upper Tocantins River - Porto Nacional, Tocantins, Brazil; and Nanay River - Iquitos, Peru (Figure 1).

### Ethics Statement

No statement from an ethics committee was necessary, and the manuscript did not involve any endangered or protect species. All samples were extracted from dead specimens collected with appropriate permissions under authorization number 22512 issued by SISBIO/Instituto Chico Mendes de Conservação da Biodiversidade. We used the ice-slurry method for killing following [33] as they are tropical warm water species and the collected specimens are all smaller than 5 cm SL. All specimens were preserved in alcohol. The reported localities do not include protected areas.

### Nomenclatural Acts

The electronic version of this document does not represent a published work according to the International Code of Zoological Nomenclature (ICZN), and hence the nomenclatural acts contained in the electronic version are not available under that Code from the electronic edition. Therefore, a separate edition of this document was produced by a method that assures numerous identical and durable copies, and those copies were simultaneously obtainable (from the publication date noted on the first page of this article) for the purpose of providing a public and permanent scientific record, in accordance with Article 8.1 of the Code. The separate print-only edition is available on request from PLoS by sending a request to PLoS ONE, 1160 Battery Street, Suite 100, San Francisco, CA 94111, USA along with a check for $10 (to cover printing and postage) payable to “Public Library of Science”.

In addition, this published work and the nomenclatural acts it contains have been registered in ZooBank, the proposed online registration system for the ICZN. The ZooBank LSIDs (Life Science Identifiers) can be resolved and the associated information viewed through any standard web browser by appending the LSID to the prefix “http://zoobank.org/”. The LSID for this publication is: urn:lsid:zoobank.org:pub:033A323A-18F7-4788-8405-32D78BF65B13.

### Morphological Analyses

Specimens from all three localities were cleared and stained following the methodology of [34].

### Molecular Analyses

The molecular systematic analyses used newly determined sequences obtained from the mitochondrial barcode marker COI as well as previously published sequences obtained from the NCBI and BOLD databases.

For the newly determined sequences, a fragment of epaxial musculature was submitted to the standard protocol for DNA extraction and purification from the Qiagen QIAamp DNA FFPE Tissue kit. The fragments were amplified and sequenced using the primers VF2_t1 and FishR2_t1 [35]–[37]. All primers were appended with M13 tails on sequencing reactions. The PCR profile consisted of 2 min at 95°C, 35 cycles of 30 sec at 94°, 40 sec at 52°C, and 1 min at 72°C, with a final extension step for 10 min at 72°C. Sequencing reactions were performed with the use of the BigDye® Terminator v.3.1 Cycle Sequencing kit (Applied Biosystems, Inc.), with 25 cycles of 10 sec at 95°C, 5 sec at 50°C and 4 min at 60°C. Sequencing products were processed in an ABI 3500 capillary system (Applied Biosystems, Inc.).

The chromatograms were checked and aligned using the BioEdit 7.053 [38] software with its built-in ClustalW routine [39]. The alignment was visually inspected for accuracy and to minimize missing data. All the newly determined sequences are available at the BOLD database (http://www.barcodinglife.com) under the project acronym PUFER. The GenBank accession numbers for all newly determined and previously published sequences used in the present manuscript are summarized in Table 1. The dataset consisted of a 651 bp COI matrix, and we used the MEGA 5.06 software [40] to determinate the TN93+G+I as the most appropriate model of sequence evolution based on the Akaike criterion (AIC) [41].

**Table 1:** Taxonomic sampling and accession numbers.

Taxon	Accession No.	Taxon	Accession No.
**Família Triodontidae**			JQ841396
*Triodon macropterus*	AP009170		JQ840304
**Família Diodontidae**			GU225449
*Diodon holocanthus*	AP009177	*Sphoeroides annulatus*	GU440524
*Chilomycterus reticulatus*	AP009188	*Sphoeroides testudineus*	KC959927*
**Família Tetraodontidae**			KC959928*
*Lagocephalus laevigatus*	AP011934		GU225665
	JQ365394		GU225453
	JQ365392		GU225450
	JQ365395		JQ842706
	JQ365393		JQ843064
	KC959926*		JQ840306
*Lagocephalus inermis*	FJ434549		GU225664
	GU804920		JQ365576
*Lagocephalus lunaris*	DSFSG9111		JQ365575
*Lagocephalus lagocephalus*	AP011933		JQ365574
*Lagocephalus guentheri*	HQ149858		GU440524
	JF493722	*Colomesus psittacus*	KC959923*
	JF493724		KC959924*
*Lagocephalus wheeleri*	JF952772		KC959925*
	AP009538	*Colomesus asellus*	KC959904*
	FJ434551		KC959907*
*Lagocephalus spadiceus*	EU595163		KC959908*
	EU595161		KC959909*
*Takifugu ocellatus*	AP009536		KC959910*
*Takifugu poecilonotus*	AP009539		KC959911*
*Takifugu snyderi*	AP009531		KC959913*
*Takifugu oblongus*	AP009535		KC959914*
*Takifugu pardalis*	AP009528		KC959915*
*Takifugu niphobles*	AP009526		KC959916*
*Takifugu porphyreus*	AP009529	*Colomesus tocantinensis*	KC959905*
*Tetraodon biocellatus*	KC959929*		KC959906*
*Tetraodon nigroviridis*	KC959930*		KC959912*
*Sphoeroides pachygaster*	EU074597		KC959917*
	EU074598		KC959918*
	EU869843		KC959919*
	CSFOM07310		KC959920*
	JF494544		KC959921*
	JF494541		KC959922*
	EU869842		
	EU869839		
	AP006745		
*Sphoeroides greeleyi*	JQ365572		
*Sphoeroides nephelus*	JQ842698		
	JQ842695		
	JQ842699		
*Sphoeroides spengleri*	JQ842704		
	JQ842695		
	JQ842701		
	JQ841395		

(*)Sequences newly determined in this study.

The neighbor-joining (NJ) and maximum-likelihood (ML) trees that encompass the genera *Triodon*, *Diodon*, *Chilomycterus*, *Lagocephalus*, *Tetraodon*, *Takifugu*, *Sphoeroides*, and *Colomesus*, were constructed using the MEGA 5.06 software [40].

The neighbor-joining sequence divergences were calculated based on the Kimura Two Parameter (K2P) distance model [42] on BOLD workbench and MEGA 5.06 software [40]. The haplotype determination was carried with the use of the server FaBox (http://birc.au.dk/software/fabox/).

## Results and Discussion

**Figure 2:**
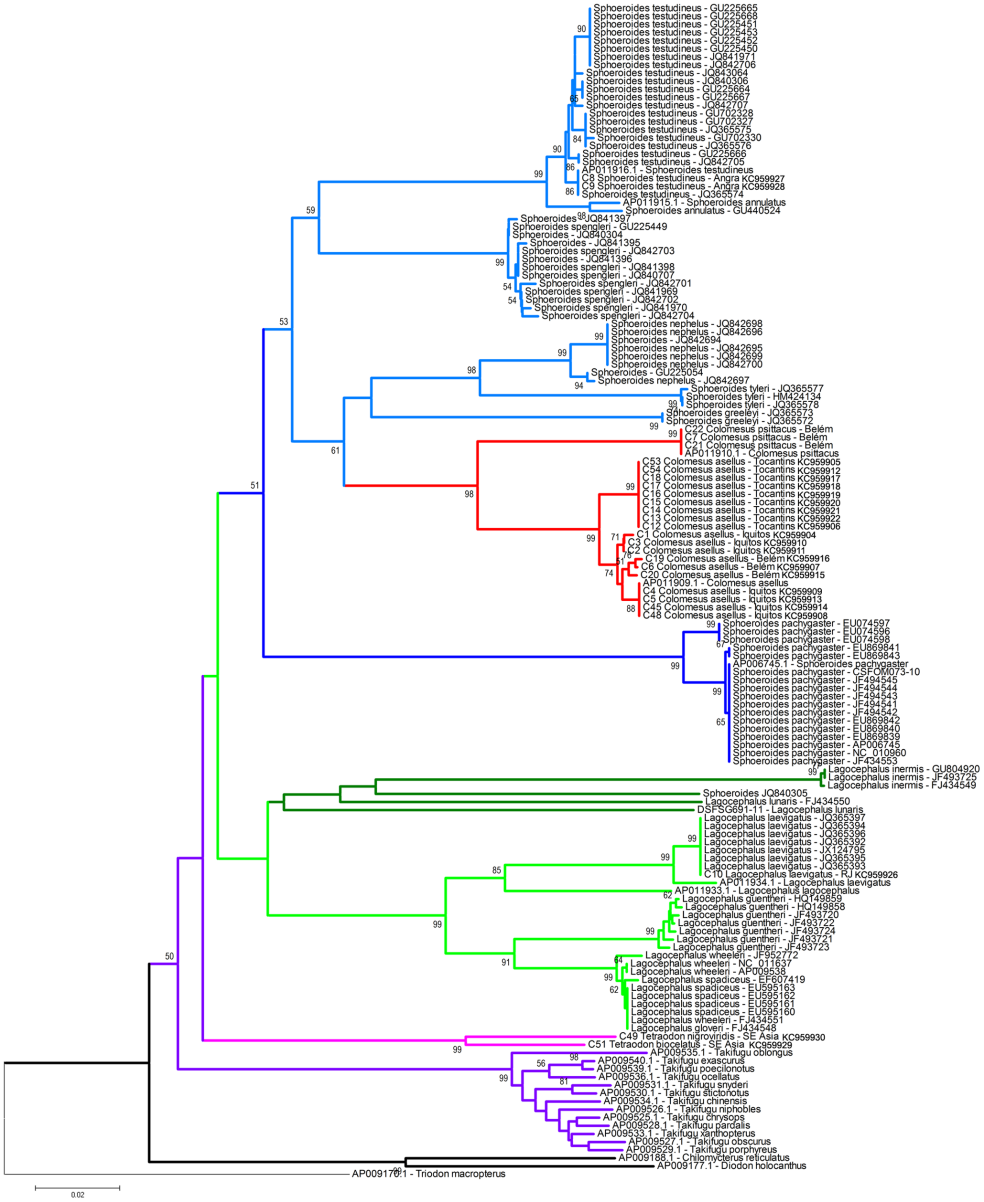
Neighbor-Joining tree based on the barcode region of the COI. The numbers near the branches represent bootstrap probabilities higher than 50%.

**Figure 3:**
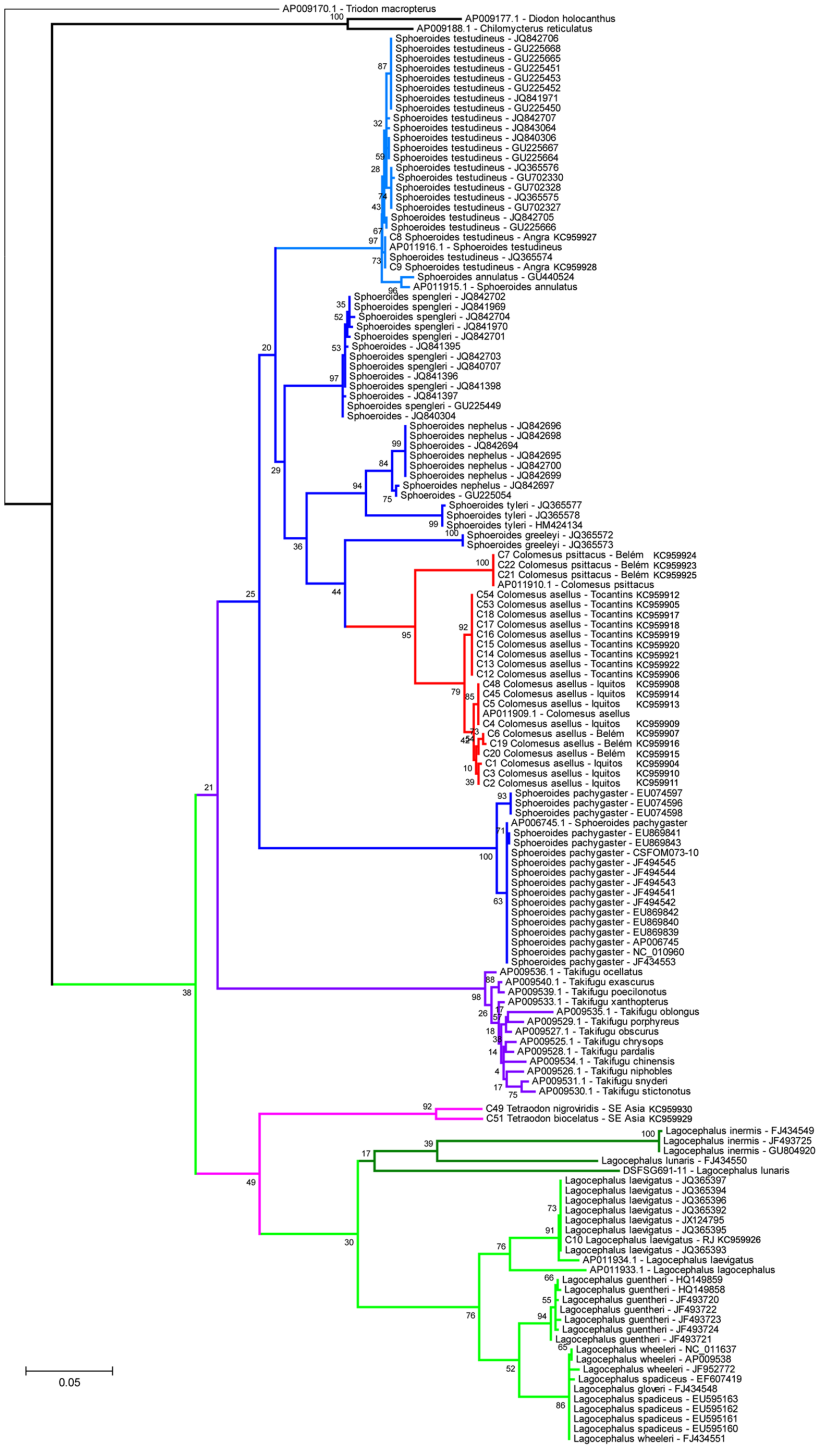
Maximum-likelihood phylogeny based on the barcode region of the COI marker. The numbers near the branches represent the bootstrap probabilities.

The neighbor-joining (NJ) and maximum-likelihood (ML) result trees are presented in Figures 2 and 3, respectively. The genus *Colomesus* was recovered as monophyletic inside the group formed by the sampled *Sphoeroides* species, in except for *Sphoeroides pachygaster. Lagocephalus* was recovered in a basal phylogenetic position in relation to *Sphoeroides* and *Colomesus*, therefore corroborating recent results such as those presented by [43]–[45].

*Colomesus* was recovered deep inside the group formed by the remaining *Sphoeroides* species, therefore suggesting *Sphoeroides* as paraphyletic, with *S*. *pachygaster* being recovered as basal in relation to all the remaining *Sphoeroides* species in all the analyses. Additionally, *Colomesus* was also recovered as the sister-taxa of the group formed by the species *Sphoeroides nephelus*, *S. tyleri*, and *S. greeleyi* in the NJ result, although it was recovered as the sister-taxa of *S. greeleyi* in the ML results.

In the same way, *Colomesu*s is clearly distinguishable from the group formed by all the *Sphoeroides* species mainly by the banded pigmentation pattern present in all the *Colomesus* species; the presence of two lateral lines, with the ventral line running the full length of the caudal peduncle; and the absence of an upraised horizontal ridge of skin ventrolaterally along the caudal peduncle. The color pattern was used by [46], along with pectoral fin ray counts, the presence of a dark bar underside of caudal peduncle, and the presence of dermal flaps across the chin, to distinguish between what at that time were considered to be the only two species of the genus, the marine/estuarine *C. psittacus*, and the freshwater *C. asellus*. The presence of a dark bar on the underside of the caudal peduncle is a prominent feature for specimens of *C. asellus* from Iquitos, but this bar is present or not in specimens from both Belém and Tocantins. Dermal flaps were observed in all specimens from both Iquitos and Belém, but such flaps were not observed in any of the examined specimens from the Tocantins drainage.

### DNA Barcode and Deep Sequence Divergence

COI amplicons were obtained from all the specimens included in the analyses. The obtained sequences clearly identified both previous accepted *Colomesus* species (*C. asellus* and *C. psittacus*), therefore being in accordance with the previous morphological diagnose presented by [46].

**Figure 4:**
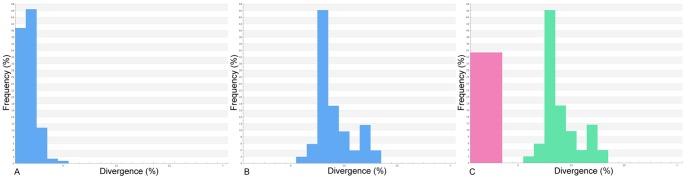
Distribution of K2P distances (%) for COI: A) within species; B) within genera; C, normalized distribution of K2P distance (%) within species. The analyses included the following taxa: *Tetraodon nigroviridis*, *Tetraodon biocellatus*, *Sphoeroides testudineus*, *Lagocephalus laevigatus*, *Colomesus asellus*, *Colomesus psittacus*, and the freshwater *Colomesus* from the Tocantins drainage.

The K2P divergence distances between congeneric species ranged from 5.557% to 12.394% with a mean distance of 8.546%, while the uncorrected K2P distance ranged from 0 to 4.472% within species. The mean K2P distance within the analyzed populations was 0.657% and the mean normalized distance within species is 1.079% (Figure 4).

**Figure 5:**
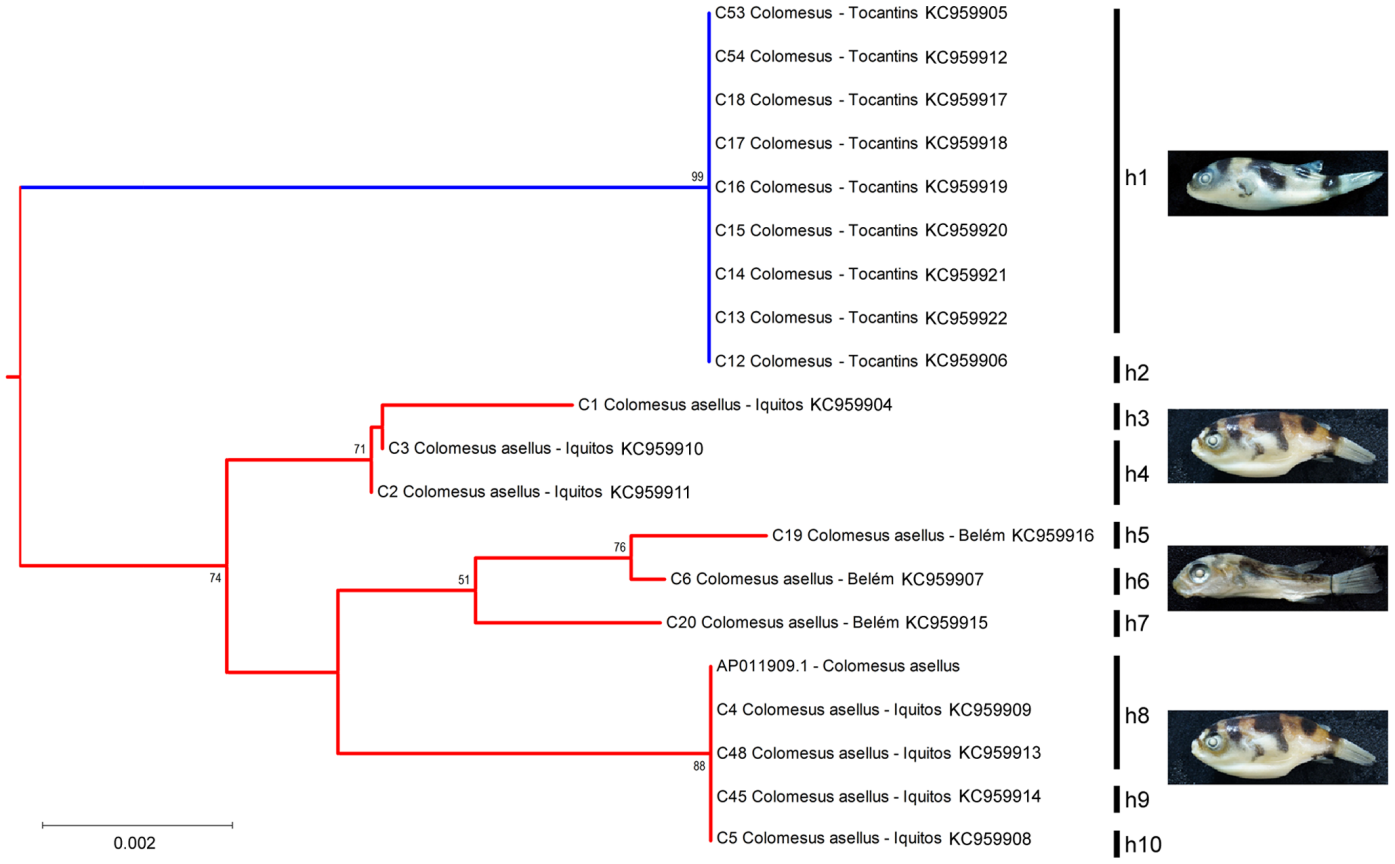
Neighbor-Joining phylogeny of the freshwater *Colomesus* and haplotype determination.

Deep sequence divergence was observed regarding the freshwater *Colomesus* from the Tocantins drainage (Figure 5). The mean sequence divergence of the specimens from both Belém and Iquitos was estimated at 1.079%, while the Tocantins distances ranged from 1.955% to 3.063%, with a mean distance of 2.166%. The observed sequence divergence values together with the congruence observed from both molecular and morphological phylogenetic approaches used here suggest the existence of an overlooked species within the genus *Colomesus*.

**A new** ***Colomesus*** **species from the Tocantins River, Brazil**

#### Systematics

**Tetraodontiformes** *sensu* Tyler, 1980 [47]

**Tetraodontidae** *sensu* Santini & Tyler, 2003 [45]

***Colomesus*** Gill, 1885 [2]

***Colomesus tocantinensis*** **nov. sp.** urn:lsid:zoobank.org:act:9B8ACCB5-FF55-4514-901B-6366FB6EA307

#### Derivation of name

The specific epithet *tocantinensis* refers to the type locality, Porto Nacional, State of Tocantins, Brazil.

#### Holotype

**Figure 6:**
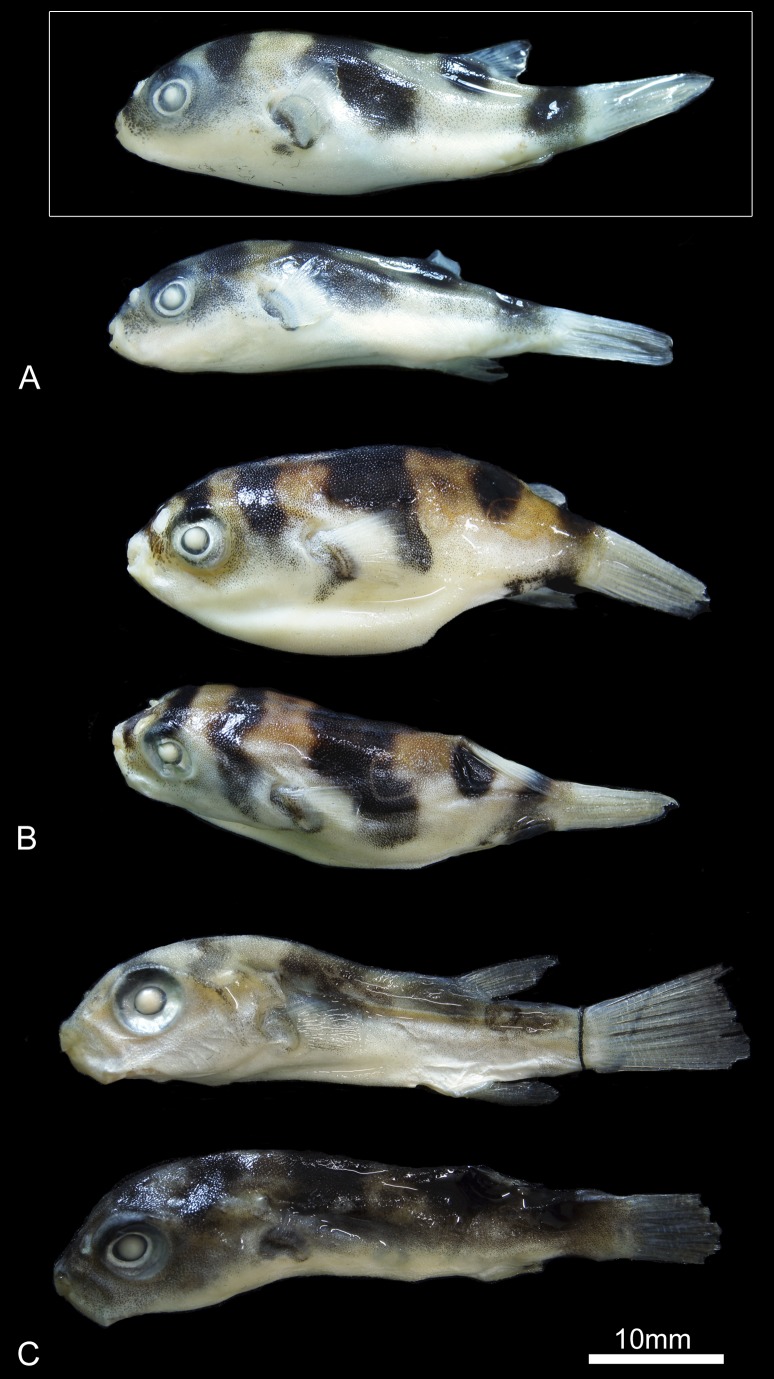
External morphology of the genus *Colomesus*. A) *Colomesus tocantinensis* **nov. sp.** – Tocantins (holotype PNT.UERJ.405 highlighted in white); B) *Colomesus asellus* – Iquitos; C) *Colomesus asellus* – Belém.

PNT.UERJ.405 (Figure 6).

#### Paratypes

PNT.UERJ.396, PNT.UERJ.397, PNT.UERJ.398, PNT.UERJ.399, PNT.UERJ.400, PNT.UERJ.401, PNT.UERJ.402, PNT.UERJ.403, PNT.UERJ.404.

#### Type-locality

The specimens are from the Tocantins River near Porto Nacional, State of Tocantins, Brazil.

#### Diagnosis

*Colomesus* species diagnosed by six to seven basal pterygiophores and nine rays in the anal fin (*contra* ten to eleven in both *C. asellus* and *C. psittacus*); ten basal pterygiophores and rays in the dorsal fin (*contra* eleven for both *C. asellus* and *C. psittacus*); the absence of dermal flaps across the chin (*contra* its presence uniquely in *C. asellus*); a caudal peduncle with eight vertebrae; and an opercle with a posterior ventral border subdivided in a ventral and a posterior region, the herein called “inverted V” shape (*contra* the triangular opercle exhibited by both *C. asellus* and *C. psittacus*).

#### Description

The holotype (PNT.UERJ.405) is 29,62 mm SL (Figure 6), with 10,37 mm HL; the entire type-series ranges from 27.02 mm to 34.9 mm SL. The meristic and morphometric data of the type series is presented in Table 2. The extent of the dorsal and ventral lateral lines is similar to those found in *C. asellus*. The prickles extend along the dorsal, lateral, and ventral surfaces of the body, from the level of the eye to the origin of the dorsal fin.

**Table 2:** Morphometric and meristic data of the type series of *Colomesus tocantinensis* nov. sp.

Register	SL	HL	PR	DR	AR	CR	IOL
**PNT.403**	30.84	10.75	15	10	9	11	5.8
**PNT.404**	34.9	11.83	15	10	9	11	5.9
PNT.405*	29.62	10.37	16	10	9	11	5.47
**PNT.395**	30.35	11.25	15	10	9	11	6.35
**PNT.396**	29.28	11.1	16	9	9	11	6.78
**PNT.397**	29.59	10.79	16	10	9	11	5.48
**PNT.398**	29.46	10.6	15	10	9	11	5.77
**PNT.399**	30.66	11.99	16	10	9	11	5.55
**PNT.400**	32.92	11.75	15	10	9	11	6.03
**PNT.401**	27.02	9.61	16	10	9	11	5.12

SL, standard length; HL, head length; PR, pectoral fin rays; DR, dorsal fin rays; AR, anal fin rays; CR, caudal fin rays; IOL, interorbital length.

(*)Holotype.

The color pattern of *Colomesus tocantinensis* **nov. sp.** is essentially the same as that of *Colomesus asellus,* with five transverse dark bars across the dorsal region of the body. A dark blotch on the underside of the caudal peduncle, which is a state used by [46] to diagnose *Colomesus asellus, is* present or absent, being vestigial to unobservable or absent in several specimens. The interspaces between the dark bars are light yellow, with gradually decreasing pigmentation and becoming white in the ventral region (Figure 6). However, the light yellow to pale pattern presented by *C. tocantinensis* **nov. sp**. clearly contrasts with the gold-yellow pattern present in specimens from Iquitos and Belém.

The nasal sac is higher than that presented in the specimens of *C. asellus*. Two large lateral and anteromedial nostrils are present. They are similar to those found on *C. psittacus*, rather than the two small nostrils exhibited by *C. asellus*. The anterior surface of the nasal sac is smooth while the posterior surface of it is folded as in *C. psittacus*, exhibiting a “T-shaped” ridge with a relatively small dorsal flap. This flap seems much smaller than the one found on *C. asellus,* although more flexible when compared to *C*. *psittacus*.

The presence of dermal flaps across the chin is another character used by [46] to distinguish *C. asellus* from *C. psittacus*. No dermal flaps could be seen in the examined specimens from the Tocantins River, although they are always present in examined specimens from Iquitos and Belém.

**Figure 7:**
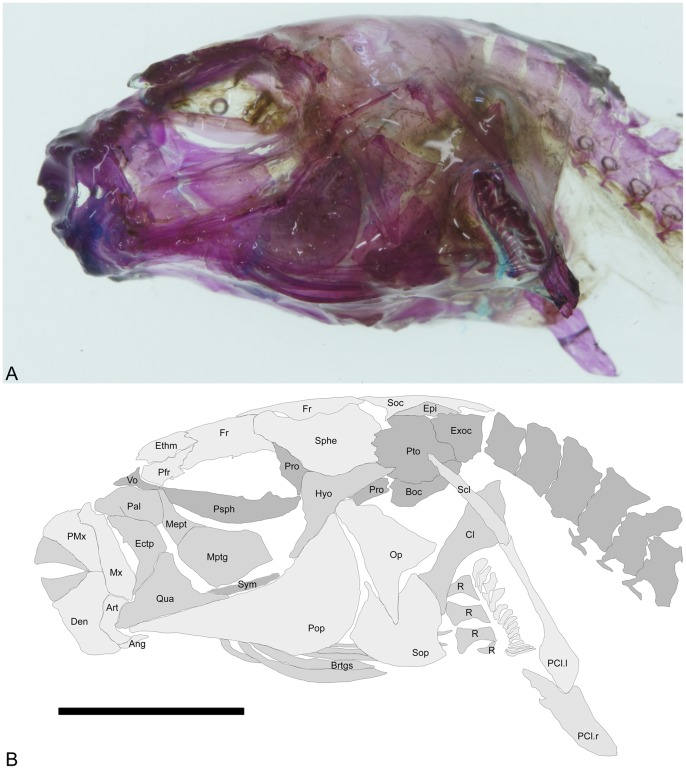
*Colomesus tocantinensis* nov. sp. (PNT.UERJ.398). A) left photograph of the head; B) anatomical interpretations. Abbreviations: Ang, angular; Art, articular; Boc, basioccipital; Brstgs, branchiostegals; Cl, cleithrum; Den, dentary; Epi, epiotic; Ethm, ethmoid; Exo, exoccipital; Fr, frontal; Hyo, hyomandibula; Ecptg, ectopterygoid; Mept, mesopterygoid; Mtptg, metapterygoid; Mx, Maxilla; Op, opercle; Pal, palatine; PCl.l/r, ventral post-cleithrum left and right; Pfr, prefrontal; PMx, premaxilla; Pop, preopercle; Pro, prootic; Psph, parasphenoid; Pto, pterotic; Qua, quadrate; R, radials; Scl, supracleithrum; Soc, supraocciptal; Sop, subopercle; Sphe, sphenotic; Sym, sympletic; Vo, vomer. Scale bar equals 5 mm.

**Figure 8:**
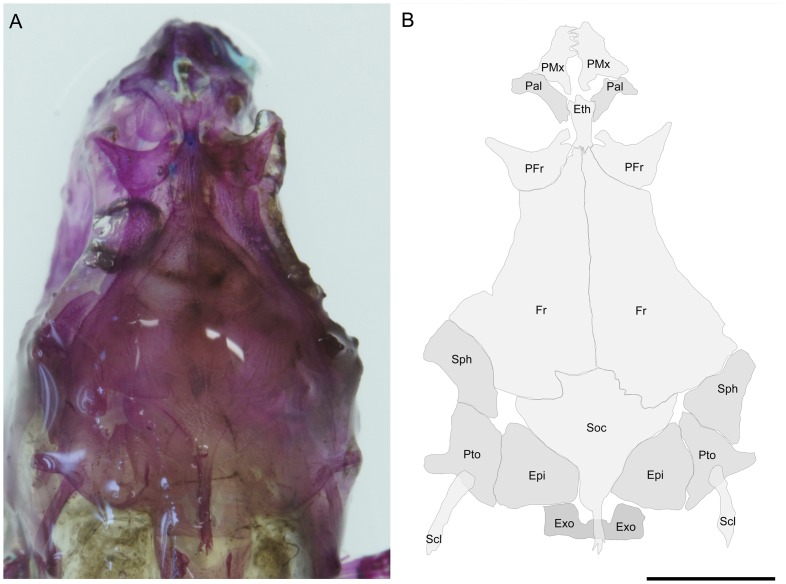
*Colomesus tocantinensis* nov. sp. (PNT.UERJ.398). A) top photograph of the head; B) anatomical interpretations. Abbreviations: Epi, epiotic; Ethm, ethmoid; Exo, exoccipital; Fr, frontal; Pal, palatine; Pfr, prefrontal; PMx, premaxilla; Pto, pterotic; Scl, supracleithrum; Soc, supraocciptal; Sphe, sphenotic. Scale bar equals 5 mm.

**Figure 9:**
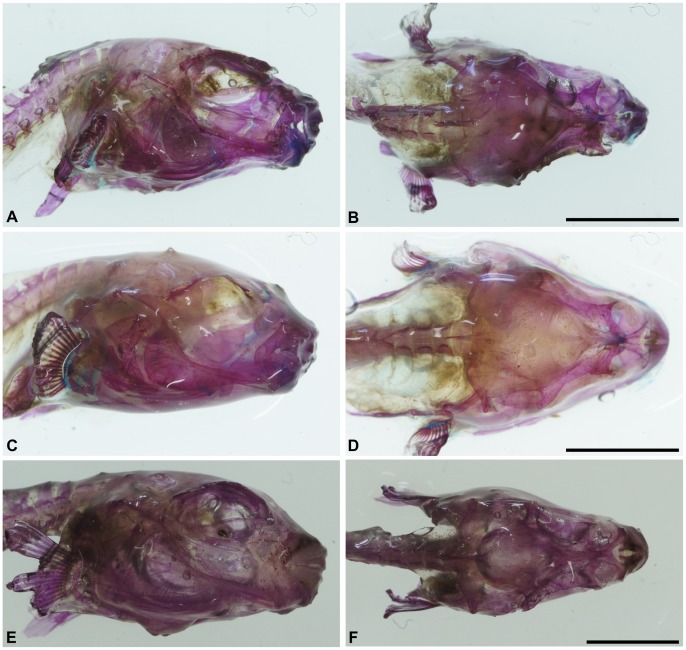
Right and top photographs from cleared-and-stained specimens of: A–B) *Colomesus tocantinensis* nov. sp. – Tocantins (PNT.UERJ.398); C–D) *Colomesus asellus* – Iquitos (PNT.UERJ.470); E–F) *Colomesus asellus* – Belém (PNT.UERJ.386).

The skull is partially similar to those found in *Colomesus asellus* described and figured by [46], although the frontals exhibit a wide posterior border and prominently participate in the orbital margin (Figures 7–9). The prefrontals are triangular and articulate medially with the ethmoid, which posteriorly articulates with the frontals and anteriorly with the palatines (Figure 8). The supraoccipital is roughly triangular and well developed, with an elongate posterior process which covers the first vertebrae (Figure 8). The sphenotics articulate postero-laterally with the frontals and, in the examined specimens, they neither contact nor closely approach the prefrontals. The lateral wing of the sphenotics is only partially developed (Figure 8). Posterior to the sphenotics, the pterotics (Figures 7 and 8) articulates posteriorly with the slender supracleithrum and medially with the epiotics, which articulates medially with the supraoccipital (Figure 9).

**Figure 10:**
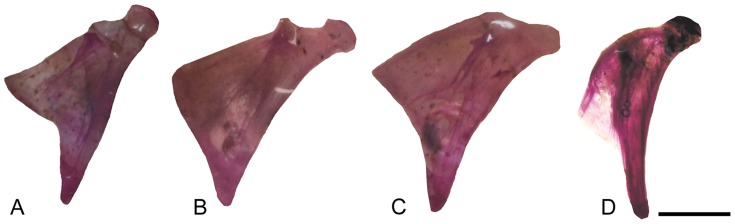
Isolated opercles from: A) *Colomesus tocantinensis* nov. sp. – Tocantins (PNT.UERJ.398); B) *Colomesus asellus* – Iquitos (PNT.UERJ.470); C) *Colomesus asellus* – Belém (PNT.UERJ.386); D) *Colomesus psittacus* – Belém (PNT.UERJ.387). Scale bar equals 1

In lateral view, the skull is characterized by the wide preopercle with about 110 degrees between both horizontal and vertical rami (Figure 7), with the preopercular canal running along its anterior border, and by the opercle which is divided in two distinct regions, having ventral and posterior wings, the herein called “inverted V” shape, distinct from the condition found in all other examined specimens of *Colomesus* (Figure 10). The subopercle is sturdy, with two small dorsal processes.

The parasphenoid is elongate and does not exhibit any developed dorsal flange (Figures 7 and 9). The hyomandibula is roughly triangular and has a slender ventral region; its wide head articulates dorsally with the sphenotics, and its upper posterior edge with the anterior end of the opercle (Figure 7).

The palatine is wide and somewhat triangular, with a robust anterior process for the maxilla (Figure 7). The maxilla is robust, with an anterodorsal region articulating with the premaxilla and a posterior expanded region, medially concave for muscle insertion. The ectopterygoid articulates dorsally with the palatine and ventrally with the anterodorsal border of the quadrate. The metapterygoid is wide and composes almost the entire ventral orbital region (Figure 7). It articulates anteriorly with the mesopterygoid (Figure 7), and with the posterior end of the large and triangular quadrate (Figure 7). The quadrate exhibits a well-developed posteroventral spine articulating posteriorly with the slender symplectic (Figure 7), and anteriorly with the articular. The articular is “L” shaped and articulates anteriorly with the robust dentary and ventrally with the small angular (Figure 7).

**Figure 11:**
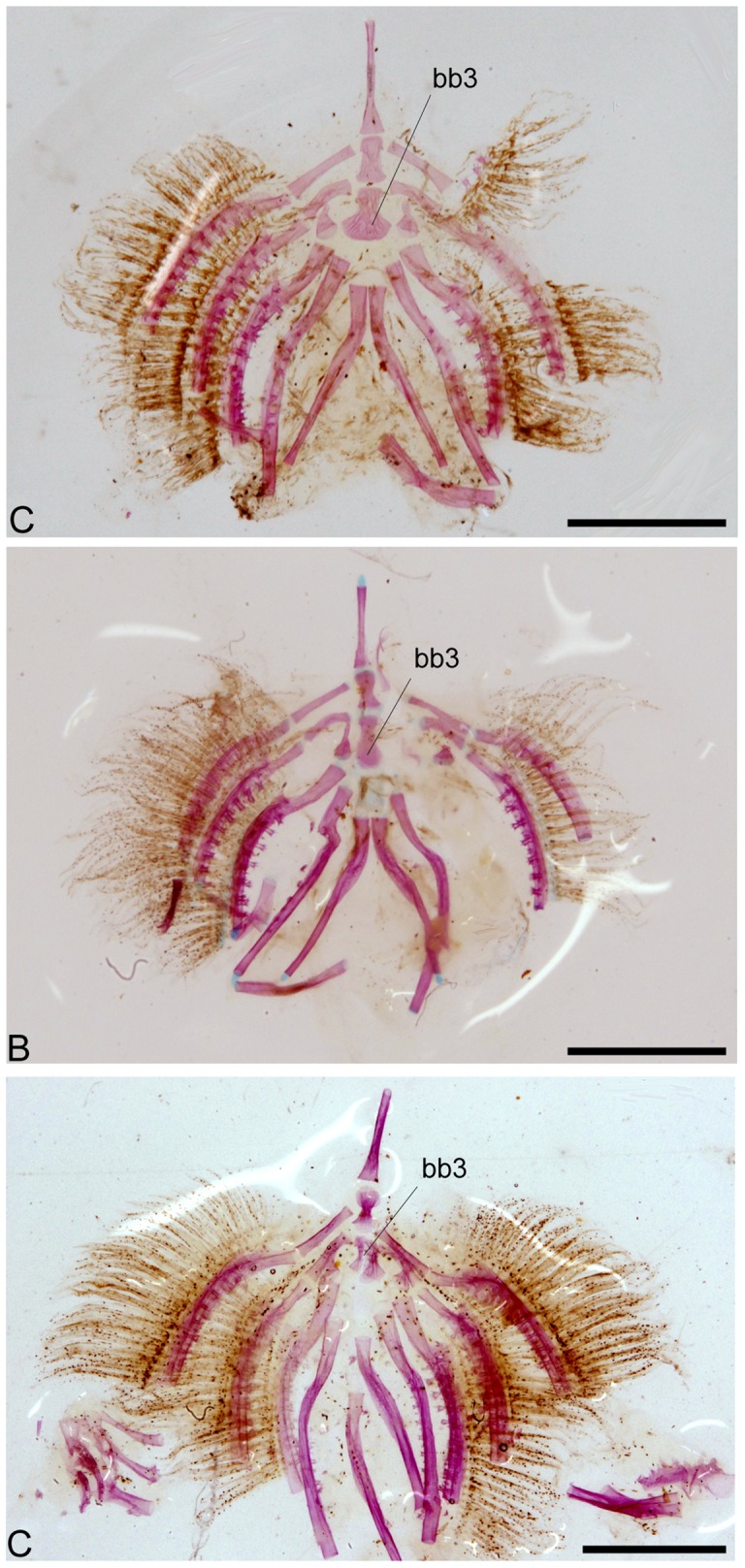
Isolated branchial apparatus from: A) *Colomesus tocantinensis* nov. sp. – Tocantins (PNT.UERJ.404); B) *Colomesus asellus* – Iquitos (PNT.UERJ.470); C) *Colomesus asellus* – Belém (PNT.UERJ.386). Scale bar equals 2.5

Five branchiostegal rays (Figure 7) are present and the branchial apparatus is strikingly similar in all the examined specimens (Figure 11).

The pectoral girdle is robust and formed by a wide cleithrum, somewhat triangular and posteriorly expanded, articulating dorsally with the slender supracleithrum. The supracleithrum articulates ventrally with the two postcleithra; a slender dorsal postcleithrum, followed by the posteriorly expanded ventral postcleithrum (Figure 7). There are four radials and sixteen pectoral fin rays (Figure 7).

**Figure 12:**
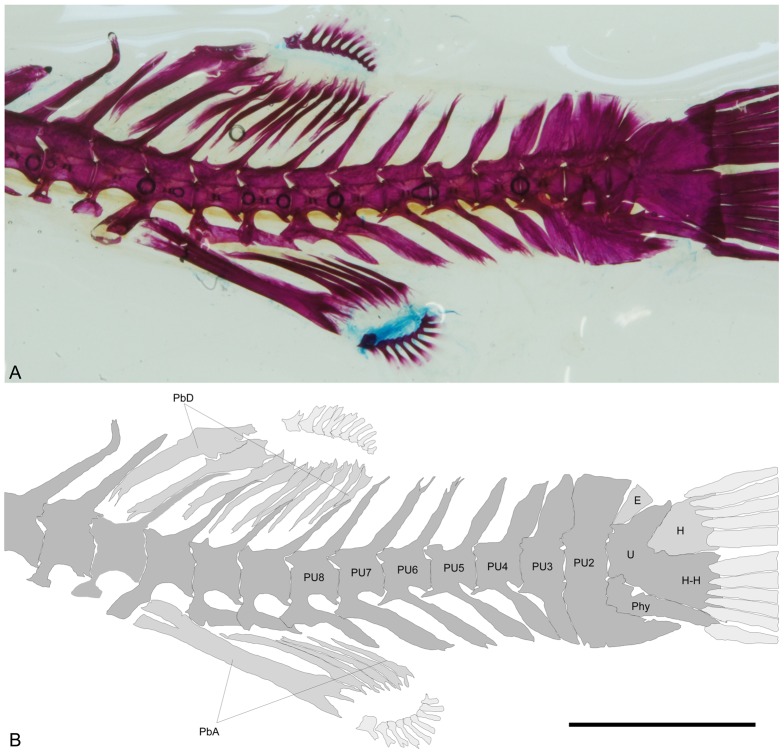
*Colomesus tocantinensis* nov. sp. (PNT.UERJ.403). A) left photograph of the unpaired fins and caudal endoskeleton; B) anatomical interpretations. Abbreviations: E, epural; H, dorsal hypural plate; H-H, ventral hypural plate fused with the ural centrum; Phy, parhypural; PU, pre-ural vertebrae; PbD, dorsal-fin basal pterigiophores; PbA, anal-fin basal pterigiophores. Scale bar equals 5 mm.

**Figure 13:**
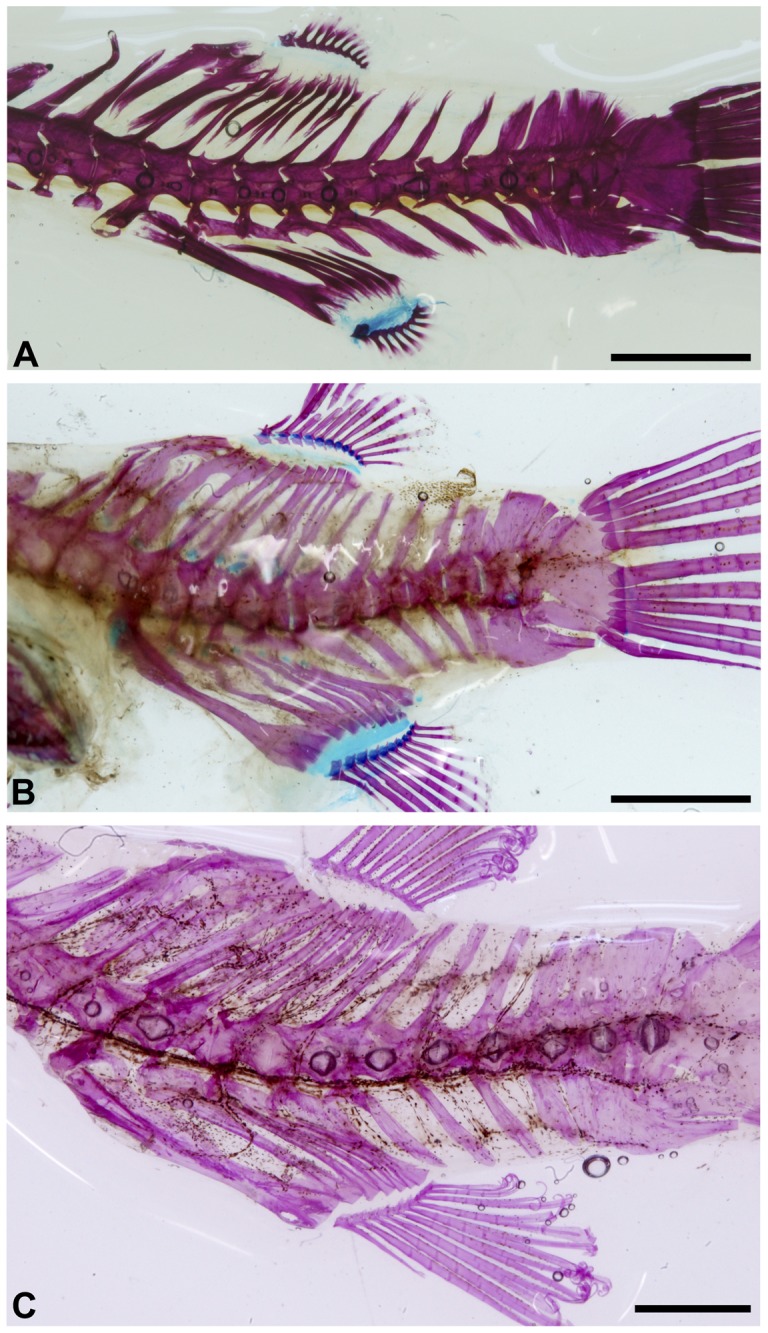
Left view photographs from cleared-and-stained specimens of: A) *Colomesus tocantinensis* nov. sp. (PNT.UERJ.403); B) *Colomesus asellus* – Iquitos (PNT.UERJ.470); C) *Colomesus asellus* – Belém (PNT.UERJ.386). Scale bar equals 5

**Figure 14:**
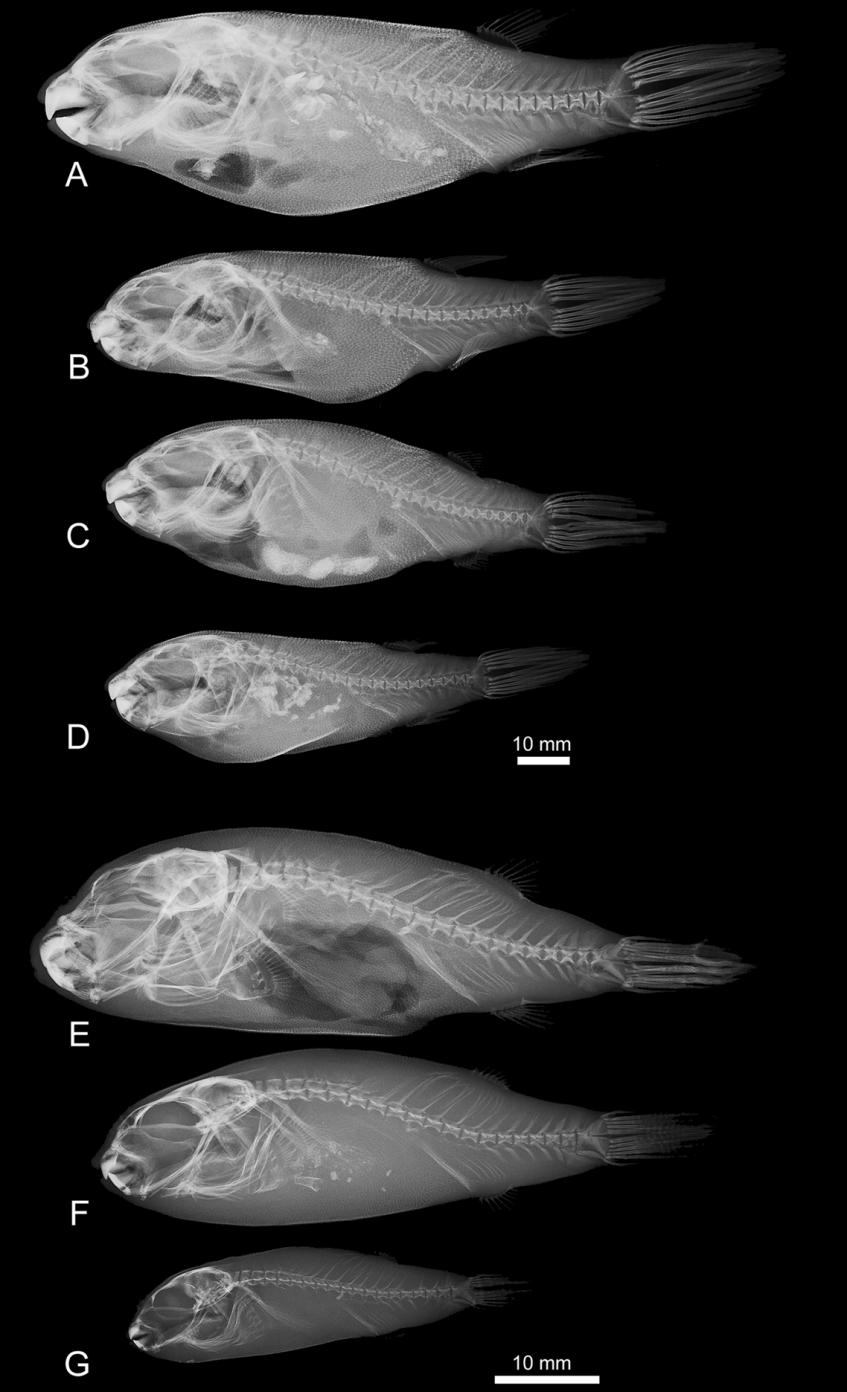
High-definition x-ray images of: A–D, *Colomesus psittacus* USNM.393077; E–G, *Colomesus asellus* USNM.191569. Scale bar equals 10

The axial skeleton has 19 vertebrae. The dorsal fin originates between vertebrae 7–8 and has ten basal pterygiophores and ten fin rays (Figures 12–14). The anal fin is located beneath the 9^th^ vertebra and has six basal pterygiophores and nine fin rays.

The caudal skeleton (Figure 12) has a wide ural centrum formed by the preural centrum 1, the ural centrum, the ventral hypural plate, and the postero-dorsal expansion which articulates anteriorly with the almost triangular epural, and posteriorly with the dorsal hypural plate (Figure 12). Eleven caudal fin rays, five dorsal and six ventral, are present in all of the specimens, both the uppermost and the two lowermost rays are unbranched.

### Phylogeography of the South American Freshwater Pufferfishes

Although the influence of marine incursions after the Miocene is still under debate, the Caribbean (or Miocene) marine incursion, via the Llanos Basin (Colombia-Venezuela), is well accepted based on both geological and paleontological evidence, suggesting that these incursions may have isolated marine taxa within the western South America freshwater environments [48]–[52]. This might be the case for the freshwater tetraodontids. As pointed by [53], this scenario predicts that the distribution of the marine sister groups of marine lineages should be related with the Caribbean or western Atlantic, the age of freshwater taxa should be coincident with marine incursions, and the biogeographic congruence should be observed among multiple unrelated taxa, conditions only partially filled by the genus *Colomesus.*

The timing of divergence between the brackish/marine *C. psittacus* and the freshwater *C. asellus* was recently discussed [45], based on a multiple loci approach including both nuclear and mitochondrial markers. The authors dated the split between 2,5-7My, therefore postdating the Miocene marine incursions usually used to explain the presence of several marine groups within the western Amazon. In this sense, as observed by [45], the colonization carried by the tetraodontids in South America could be presumably related to the Pliocene global climate oscillations. Additionally, the basal split of the *Colomesus* from Tocantins and from Iquitos/Belém agrees with the general area cladogram of neotropical fishes presented by [54] in which the Xingu/Tocantins-Araguaia group was recovered in a basal position in relation to the groups from the lowlands of Western and Eastern Amazon.

It was recently proposed [55], based on the distribution of characiforms, that recent marine incursions would have isolated fish populations in upland terrains or refuges, where lineage divergence is maximized, followed by dispersal episodes back to the lowlands. The “museum hypothesis” predicts that lowlands exhibit a higher number of species, but lower levels of endemism, than highlands, and that the upland refuges would represent areas of high endemism.

Looking on the molecular phylogeny of the serrasalmids *Pygocentrus* and *Serrasalmus*, [56] proposed a phylogenetic test which predicts that basal lineages in a phylogeny of widespread fishes would occur in highland areas, and lowland lineages would have originated only during the last 5 Ma. Additionally, [57] studying the genetics of *Symphysodon* cichlids, indicated the effects that marine incursions would have in population structure, stating that populations in upland terrains or refuges would exhibit reduced genetic variation, while populations in lowlands would represent multiple upland sources, therefore exhibiting a high level of genetic variation, and that populations in lowlands would show a demographic pattern of expansion.

Our results recovered the Upper Tocantins lineages in a basal position in relation to all the remaining specimens, with the sequences being collapsed in uniquely two haplotypes (Figure 5), the first one (h1), represented by eight sequences, and the second haplotype (h2), represented by a unique sequence. This suggests low genetic variation, at least among the studied sampling, and a history initially related with the eastern Amazon, followed by a subsequently expansion to the western South America.

### The Tocantins-Araguaia Ichthyofauna

The Tocantins-Araguaia drainage is the fourth largest Brazilian drainage, draining part of the northern end of the Brazilian shield directly to the eastern end of the Amazon Basin. It exhibits a recent geomorphological history, within a still tectonically active sedimentary basin with recent subsidence episodes, which are related with the high load of sediments observed within the basin, leading the development of the Bananal Plain, in the lower part of the drainage, mainly during the Quaternary [58]–[59].

The Tocantins drainage, specially the Upper Tocantins River, is constantly regarded as an area of high endemism, with several fish taxa restricted to this area having been described, such as *Leporinus taeniofasciatus* (Anostomidae), *Sternarchorhynchus mesensis* (Apteronotidae), *Aspidoras albater*, *A. eurycephalus* (Callichthyidae), *Acestrocephalus maculosus*, *Astyanax unitaeniatus*, *Astyanacinus goyanensis*, *Creagrutus atrisignum*, *C. britskii*, *C. mucipu*, *C. saxatilis*, *Hyphessobrycon hamatus*, *Moenkhausia tergimaculata*, *Cetopsis caiapo*, *C. sarcodes* (Cetopsidae), *Characidium stigmosum* (Crenuchidae), *Pimelodella spelaea* (Heptapteridae), *Ancistrus aguaboensis*, *A. jataiensis*, *A. minutus*, *A. reisi*, *Corumbataia veadeiros*, *Hemiancistrus micrommatos*, *Hypostomus ericae*, *Gymnotocinclus anosteos*, *Lamontichthys avacanoeiro* (Loricariidae), *Apareiodon argenteus* and *A. cavalcante* (Parodontidae), *Cynolebias griseus*, *C. notatus*, *Rivulus planaltinus*, and *Simpsonichthys marginatus* (Rivulidae), the herein described *Colomesus tocantinensis* **nov. sp.** (Tetraodontidae), and *Ituglanis bambui* and *I. mambai* (Trichomycteridae).

In the same way, [60]–[62] pointed that the Tocantins River is the only river system related with the Amazon Basin, and draining shield areas which exhibit a considerable number of fish taxa known from the lowlands of the central and western Amazon. The faunal similarity between both basins is constantly related with the evolution of the Bananal Plain as a selective barrier for the lowland fauna and the upper part of the drainage, and the list of typical lowland ichthyofauna shared by the Tocantins-Araguaia and the Amazon drainages includes, as presented by [60], *Leporinus trifasciatus* (Anostomidae), *Arapaima gigas* (Arapaimatidae), *Auchenipterichthys coracoideus* (Auchenipteridae), *Mylossoma* spp., *Pygocentrus nattereri* (Characidae), *Cetopsis candiru*, *C. coecutiens* (Cetopsidae), *Cichla monoculus*, *C. pleiozona*, *C. kelberi* (Cichlidae), *Psectrogaster amazonica* (Curimatidae), *Thorachocharax stellatus* (Gasteropelecidae), *Osteoglossum bicirrhosum* (Osteoglossidae), *Pellona castelnaeana*, *Pristigaster cayana* (Pristigasteridae), and *Colomesus asellus* (Tetraodontidae). In this sense, even still under debate, it seems clear that the Tocantins-Araguaia drainage has a composite nature including both lowland and upland ichthyofauna, as argued by [62].

## Conclusions

Based on a comprehensive analysis including both morphological and molecular methodologies using the cytochrome C oxidase I gene, we were able to discuss aspects of the phylogeny and phylogeography of the South American freshwater pufferfishes of the genus *Colomesus*.

Our molecular results based on the COI marker agrees with the recent results such as [43]–[45], and suggest that the genus *Sphoeroides* should be revised, mainly regarding the phylogenetic position recovered for the genus *Colomesus,* deeply nested within the *Sphoeroides* tree, and the basal position recovered for *S. pachygaster.* We plan further investigations along these lines to reconcile any conflicts between these molecular hypotheses presented herein and morphologically based interpretations [47] of the phylogeny of the taxa of *Colomesus*, *Sphoeroides*, and *Lagocephalus*.

The use of molecular systematic techniques together with morphological methodologies confirmed the identification of a new cryptic pufferfish species from the Upper Tocantins drainage, *Colomesus tocantinensis* **nov. sp.** Morphological features such as the color pattern, the absence of dermal flaps across the chin, the distinct ‘inverted V’ opercle shape, and the caudal peduncle morphology, all support the description of *Colomesus tocantinensis* **nov. sp.**, as a new pufferfish species from the South American freshwater drainages.

The timing of divergence between the marine/brackish species *Colomesus psittacus* and the freshwater group formed by *C. asellus* and *C. tocantinensis* **nov. sp.**, as recovered by [45], postdates the Miocene marine incursions usually used to explain the presence of tetraodontids within the Amazon freshwater environments. Therefore, it suggests that the freshwater colonization in South America, at least for the tetraodontids, could be more recent than previously expected. Additionally, together with the observed distribution of haplotypes, our results suggest that the history of tetraodontids into the Amazonian freshwater environments could be presumably related to the Pliocene global climate oscillations and its effects inside the eastern Amazon and subsequently to the western South America.

Finally, our results reinforce the Upper Tocantins drainage as an area of high endemism within the Tocantins-Araguaia drainage, although the composite nature of the entire drainage is unquestionable.
